# Early sST2 Liberation after Implantation of a Left Ventricular Assist Device in Patients with Advanced Heart Failure

**DOI:** 10.1155/2020/5826176

**Published:** 2020-12-26

**Authors:** Philipp Opfermann, Elisabeth Simader, Alessia Felli, Michele Bevilacqua, Caroline Holaubek, Martin Dworschak, Mohamed Mouhieddine, Daniel Zimpfer, Jan Hendrik Ankersmit, Barbara Steinlechner

**Affiliations:** ^1^Division of Cardiothoracic and Vascular Anesthesia and Intensive Care Medicine, Department of Anesthesia, Intensive Care and Pain Medicine, Medical University of Vienna, Vienna, Austria; ^2^Department of Surgery, Division of Thoracic Surgery, Medical University of Vienna, Vienna, Austria; ^3^Department of Surgery, Division of Cardiac Surgery, Medical University of Vienna, Vienna, Austria

## Abstract

**Background:**

The use of left ventricular assist device (LVAD) has increased considerably over the past decade; however, there is limited literature to assist in patient selection and monitoring. The frequency of adverse events remains high. We examined the early expression of circulating soluble ST2 (sST2), a biomarker with immunosuppressive and profibrotic activity, and assessed the risk of death at 1 year in patients receiving LVAD implant.

**Methods:**

We prospectively enrolled 20 heart failure patients and measured sST2, IL-33, and IL-6 serum concentrations over three weeks after LVAD implantation. We compared the kinetics of IL-6, sST2, and IL-33 release in survivors with those of nonsurvivors using mixed model two-way analysis of variance for repeated measures. We also collected data on hemodynamic parameters (i.e., cardiac output) and frequency of infections during the hospital stay.

**Results:**

LVAD therapy led to an immediate and significant improvement of the hemodynamic parameters in 1-year survivors and nonsurvivors alike. The 1-year survival rate was 65%. IL-6 concentrations showed a significant (*p* = 0.03) peak at admission to the intensive care unit following LVAD implantation, whereas sST2 levels were massively increased (*p* < 0.0003) on day 1. While 1-year survivors had persistently lower sST2 values compared to nonsurvivors during the first 3 weeks after LVAD implantation (*p* = 0.012), no differences were observed in the temporal pattern of IL-6 or IL-33. The odds of detecting *Candida* species in the bronchoalveolar lavage fluid were 14 times higher in nonsurvivors than in survivors (OR 13.7, CI 1.4-127, *p* = 0.02).

**Conclusion:**

In patients implanted with LVAD, circulating sST2 levels and frequency of *Candida* colonisation were associated with higher mortality. Awareness of this early immune response can guide physicians in risk-benefit analysis.

## 1. Introduction

Left ventricular assist devices (LVAD) are a life-saving option for patients with advanced heart failure (HF) who are not eligible, or cannot wait, for a heart transplant. LVAD support is also increasingly being used in the less ill patient population for heart tissue recovery. Patients' clinical outcomes following LVAD implantation continue to improve, with 1-year survival estimates ranging between 52 and 83% [1, 2]. However, adverse events are common and have a detrimental impact on the success of LVAD support. The implantation of an LVAD in terminal HF patients is associated with inflammation. Several studies have reported that the inflammatory milieu plays an important role in the development of early adverse events, such as multiorgan failure (MOF) [3]. LVAD implantation leads to T cell activation, heightened CD4 T cells' susceptibility to activation-induced cell death, and progressive defects in cellular immunity [4]. Infections-in particular sepsis—are still a major risk for mortality upon continuous-flow LVAD implantation [5]. After the initial trauma of heart surgery, a secondary cascade of prolonged systemic inflammation often leads to the development of systemic inflammatory response syndrome (SIRS) [6], followed by a compensatory anti-inflammatory response syndrome (CARS) [7]. The latter could explain increased susceptibility to nosocomial infections and sepsis [8]. Szerafin and coworkers reported that coronary artery bypass graft (CABG) surgery results in an “immunocompromised state” mediated by the release of soluble suppression of tumorigenicity 2 (sST2) during the first 120 hours after surgery [8]. Unlike the membrane-bound ST2, the soluble isoform of ST2 acts as a decoy receptor of IL-33 and prevents IL-33 from binding to its membrane-bound receptor. Recently, elevated sST2 levels have been associated with adverse outcomes in septic, burn, and polytrauma patients [9, 10]. Human myocytes and pneumoepithelial cells have been identified as important sources of circulating bioactive sST2 [11]. Therefore, sST2 is thought to be a marker for Th-2 cytokine-producing cells and its release signals a shift from Th-1- to Th-2-dominated immune response [12]. In clinical situations, when the immune system is confronted with a “danger” signal (e.g., myocardial infarction), circulating mononuclear cells secrete IL-1a, IL-1b, IL-6, and TNFa. In turn, this signal increases sST2 secretion in alveolar epithelial cells and cardiac myocytes, attenuating the innate and adaptive immune responses in organs exposed to environmental and autologous antigenic triggers [11]. Thus, inflammation and immunity [13, 14] trigger the interleukin-33/ST2 pathway. However, cardiomyocytes also enhance the release of IL-33 when exposed to mechanical stress. IL-33 binds to transmembrane-bound ST2 to prevent myocardial hypertrophy and fibrosis, showing “cardioprotective” properties. As sST2 acts as a decoy receptor of IL-33, the former may undermine the beneficial effects of IL-33. In fact, increased sST2 levels are associated with profibrotic remodeling and adverse outcomes in the average heart failure (HF) population [13, 15]. Therefore, sST2 has been established as a guideline-endorsed biomarker of cardiovascular risk [16, 17], along with natriuretic peptides, to assess heart failure (HF) in acute and ambulatory settings.

Furthermore, the low biological variation of sST2 makes it a potentially useful biomarker to measure serially [18] and monitor LVAD patients. Recently, in end-stage HF patients on LVAD support, it has been shown that elevated sST2 levels at baseline normalize three months after implantation [13]. However, no data is available on the temporal pattern of sST2 and IL-33 levels immediately after LVAD implantation.

This prospective observational study is aimed at characterizing the early immune response and exploring its prognostic capability for the 1-year survival after LVAD implantation. We examined sST2 and IL-33 release during the early postoperative period (i.e., first 3 weeks), in the context of other inflammation parameters such as IL-6 and C-reactive protein as well as routinely gathered hemodynamic parameters (i.e., cardiac output (liter/min), mixed venous saturation (SvO2) in %, and systolic pulmonal arterial pressure (mmHg)) measured by the Swan-Ganz Catheter during the first postoperative days. Furthermore, we evaluated the presence of pathogens in this early phase of LVAD implantation. We hypothesize that LVAD implantation induces an enhanced sST2 immune response in the early postoperative phase that may be predictive for adverse outcomes following LVAD implantation.

## 2. Materials and Methods

This prospective longitudinal trial was performed at the General Hospital Vienna, a tertiary care center, after approval by the ethical review committee of the Medical University of Vienna (EK-Nr: 1625/2013).

### 2.1. Study Population

Patients with terminal HF, irrespective of etiology, ≥18 years of age requiring LVAD (either as “bridge to transplant,” “bridge to candidacy,” “bridge to recovery,” or “destination therapy”), have been prospectively enrolled after signing the written informed consent. Patient blood routinely collected for standard blood tests during morning hours was used throughout. Measurements were taken at 11 time points: preoperatively, immediately after admission to the ICU following LVAD implantation, daily between days 1 and 7, and again on days 14 and 21. Baseline characteristics (e.g., age, sex, INTERMACS grade, and comorbidities) were collected for each patient. The patients' clinical course was followed until the first postoperative year, based on hospital and outpatient records. Additionally, the incidence of infections and the isolated pathogens have been reviewed for each patient during the hospital stay.

### 2.2. Technique of Device Implantation and Decision-Making for Type of Device

The choice of a HeartWare® (HVAD) or Thoratec® HeartMate II (HM II) device implantation as well as the implantation strategy was left at the discretion of the attending surgeon and was done according to surgical SOPs following international recommendations. Whenever possible, our teams of surgeons strive after a minimal invasive approach of LVAD implantation via bilateral minithoracotomy in HVAD or subcostal incision and right minithoracotomy in HM II as described elsewhere in detail [19]. A full sternotomy approach remained hereby reserved for postcardiotomy patients and patients with a history of left or right thoracotomy.

### 2.3. Hemodynamic Data

The cardiac output (CO, in liters/minute), the mixed venous saturation (SvO2, in %), the central venous pressure (CVP, in mmHg), and the systolic pulmonal arterial pressure (sysPAP, in mmHg) were obtained from the routinely utilized continuous-cardiac-output (CCO) Swan-Ganz Catheter (Edwards Vigilance II®) during the first 3 postoperative days. The daily maximum values of CO and SvO2 and the daily minimum values of CVP and sysPAP were collected.

### 2.4. Cytokine Analysis

Cytokine levels were measured via enzyme-linked immunosorbent assay (ELISA), using commercially available ELISA kits (DuoSet Elisa®, R&D Systems, Minneapolis, MN, USA) for sST2, IL-33, and IL-6 in accordance with the manufacturer's protocol. To minimize the risk of measurement errors, all samples were analyzed as duplicates and the respective mean values were calculated.

### 2.5. Microbiology Analysis

The biological samples were collected at the discretion of the attending ICU staff only when there was a clinical reason to do so. Different sites (lungs, blood, feces, urine, and wound) were screened depending on the location of the suspected infection. Bronchoalveolar lavage by bronchoscopy is indicated, e.g., desaturation events or suspicion of bronchopulmonary infection. Three different blood samples (from the central venous line, peripheral veins, and arterial catheter) were collected to establish aerobic and anaerobic cultures.

### 2.6. Anticoagulation Regime

Coagulation was managed using either unfractionated or low molecular weight heparin (LMWH) according to institutional guidelines (as described elsewhere in detail [20]).

### 2.7. Statistics

Patient characteristics are described using conventional summary statistics and reported as either medians with interquartile range (IQR), mean ± standard deviation, or absolute numbers (percentage), respectively. Data were screened for completeness, consistency, and outliers before analysis. Proportions were compared using Fisher's exact test or chi-square test. Risk ratios or odds ratios for dichotomous variables will be reported with 95% confidence intervals. Metric variables over time have been examined using a mixed model two-way analysis of variance for repeated measures and the Mann–Whitney *U* test for individual points. All tests were performed as two-sided tests. Differences were considered significant when *p* value was <0.05. STATA12 (StataCorp, TX) and GraphPad Prism 5 (GraphPad, San Diego, CA) were used for statistical analyses.

## 3. Results

### 3.1. Patient Characteristics and Clinical Outcome

**Figure 1:**
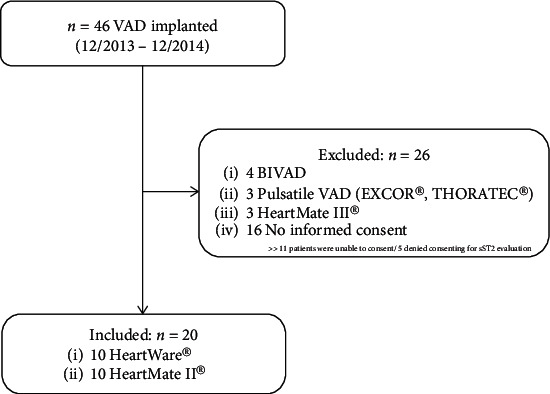
Patient flow chart. VAD = ventricular assist device; BIVAD = biventricular assist device; EXCOR®, Berlin Heart® GmbH, Berlin Germany; HeartMate II, Thoratec® Corporation, Pleasanton, CA; HeartWare® (HVAD), HeartWare International, Inc., Framingham, MA.

Twenty terminal HF patients were recruited for this study between December 2013 and December 2014 (Table 1). Ten patients received a centrifugal pump design HVAD, and ten patients received an axial flow pump HM II device (Figure 1). Detailed procedural data are indicated in Table 2. The median length of hospital stay was 41 days in 1-year survivors as well as nonsurvivors (41 (35-66) vs. 41 (33-46) n.s.). The 30-day, 90-day, and 1-year mortality rates were 0%, 20%, and 35%, respectively. One patient received a secondary extracorporeal membrane oxygenation (ECMO) device on the fifth postoperative day due to suspected right ventricular failure.

**Figure 2:**
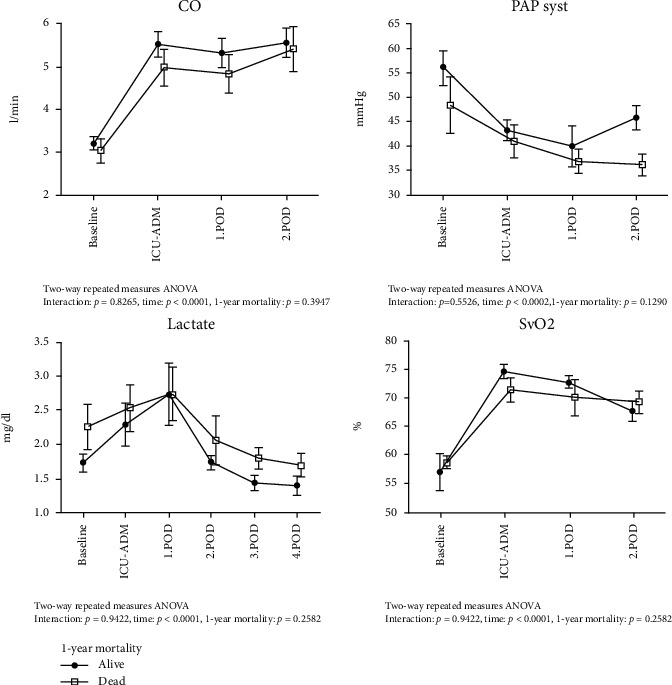
Temporal course of hemodynamic parameters during the early postoperative course in 1-year survivors and 1-year nonsurvivors. CO = cardiac output; PAP syst. = systolic pulmonal arterial pressure; SvO2 = mixed venous saturation. Hemodynamic data were obtained from the routinely utilized continuous-cardiac-output (CCO) Swan-Ganz Catheter (Edwards Vigilanz II®). Given are the results of the ANOVA mixed model analysis for repeated measures.

LVAD implantation significantly improved the hemodynamic status of 1-year survivors as well as nonsurvivors in the first postoperative week. The cardiac output (in liters/minute) (*p* < 0.0001) and the mixed venous saturation (% SvO2) (*p* < 0.0001) both increased during the first 3 days. On the other hand, the systolic pulmonal-arterial pressure (in mmHg) (*p* = 0.0002) decreased, and serum lactate values (mmol/l) normalized till the end of the first postoperative week (*p* < 0.0001). We did not observe any significant difference in the temporal pattern of hemodynamic parameters between 1-year survivors and nonsurvivors (Figure 2).

We also monitored adverse events during the hospital stay. Six patients (30%) had positive blood cultures with predominantly Gram-positive bacteria (Table 3). None of these patients met the criteria for ventricular assist device-specific or ventricular assist device-related infection [21]. The bloodstream infection was presumably central venous line-related in four patients and associated with a lower respiratory tract infection in two patients. Additionally, *Candida* species were isolated from different body sites in 11 patients (55%) during the hospital stay. The most common bacteria isolated from blood were *Staphylococcus epidermidis* and *S. haemolyticus*, whereas the most common fungal pathogens were *Candida albicans and C. glabrata* (Table 3). The frequency of bloodstream infections was not significantly different between 1-year survivors and nonsurvivors (23.1% vs. 42.9%, OR 2.5 (0.34-18); *p* = 0.61). However, *Candida* species were more frequently isolated from the bronchoalveolar lavage fluid of nonsurvivors than that of survivors (71.4% vs. 15%, OR 13.7 (1.4-127.4); *p* = 0.022). Patients with pulmonary *Candida* colonisation had numerically but nonsignificantly higher sST2 levels from postoperative day 4 onwards. The causes of death are summarized in Table 4.

### 3.2. Time Course of sST2, IL-33, IL-6, and C-Reactive Protein after LVAD Implantation

**Figure 3:**
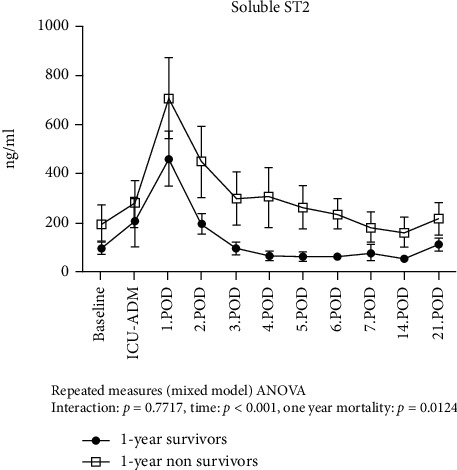
Temporal pattern of sST2 levels in 1-year survivors and 1-year nonsurvivors. Given are the results of mixed model analysis ANOVA for repeated measures.

**Figure 4:**
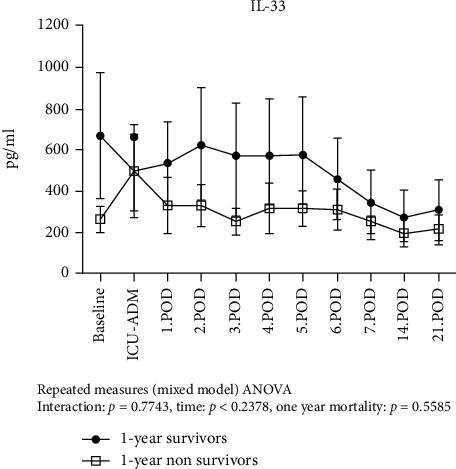
Temporal pattern of IL-33 in 1-year survivors and 1-year nonsurvivors. Given are the results of the ANOVA mixed model analysis for repeated measures.

**Figure 5:**
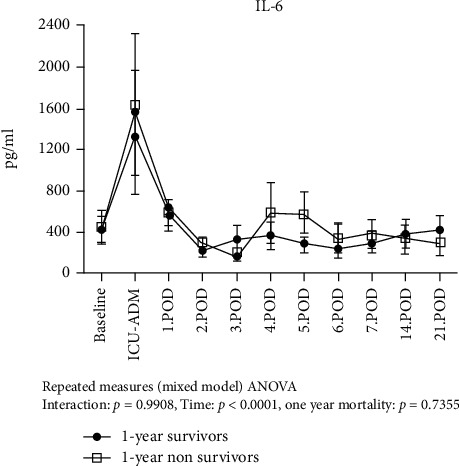
Temporal pattern of IL-6 levels in 1-year survivors and 1-year nonsurvivors. Given are the results of the ANOVA mixed model analysis for repeated measures.

We found that sST2 values significantly increased from baseline reaching concentrations > 400 ng/ml at day 1 and subsequently normalized until the end of the first postoperative week (Figure 3). Compared to the survivors, 1-year nonsurvivors had persistently higher sST2 values during this initial period (*p* = 0.012). 1-year nonsurvivors had higher preoperative baseline sST2 levels. However, this was not statistically significant (34.2 ng/ml, 95CI (0-71.6) vs. 123.6, 95CI (0-314.9); *p* = 0.123). There was no difference in IL-33 levels between survivors and nonsurvivors over the first 3 weeks post implantation (Figure 4). The IL-6 release pattern resembled that of sST2, showing an early peak at ICU admission immediately after LVAD implantation and subsequently declining till day 3. We observed no significant differences in the IL-6 temporal pattern between 1-year survivors and nonsurvivors (Figure 5). C-reactive protein levels peaked at day 2 and remained high compared to baseline values for the entire observation period. However, we did not observe any significant difference in the temporal pattern of 1-year survivors vs. nonsurvivors (*p* = 0.75).

## 4. Discussion

To the best of our knowledge, this study is the first to investigate the profile of sST2 liberation immediately after LVAD implantation in terminal heart failure patients. Our results revealed a massive enhancement of sST2 levels after LVAD implantation with a peak concentration of >400 ng/ml at day 1. Compared to 1-year survivors, nonsurvivors had persistently higher sST2 levels during the hospital stay.

The death rate in our patient cohort was 35% at 1 year, a rate comparable to that reported by previous studies [1, 2]. Circulating sST2 levels were higher than normal (mean 62.5 ng/ml; reference value < 35 ng/ml) even before implantation. Our results are supported by a previous study showing mean preoperative ST2 concentrations of 74 ng/ml in a similar patient population [13]. These findings raise the interesting possibility that preoperative sST2 levels may be used as a tool to identify which patients would benefit the most from the LVAD therapy. Although preoperative sST2 levels were higher in 1-year nonsurvivors than in survivors, the difference was not statistically significant. Still, this possibility is worth exploring further in larger trials. The concentrations of the different mediators at ICU admission suggest an initial immune response dominated by a peak of Th-1 cytokine IL-6 triggered by surgical trauma of LVAD implantation. On day 1, the massive release of sST2 points to a shift from a Th-1- to a Th-2-based immune response. Interestingly, Szerafin and colleagues showed a similar kinetic of sST2 levels after coronary artery bypass graft (CABG) surgery in patients with almost normal left ventricular function. It was then postulated that the ensuing sST2-mediated immunosuppression made these patients more susceptible to local and systemic infections [8]. Similarly, Hacker and colleagues showed that sST2 levels increase while IL-33 levels decrease immediately after extensive burn injury. The authors concluded that sST2 is involved in a transition from a proinflammatory to an immunosuppressive state by binding to IL-33 and preventing IL-33 signaling. Also in that study, higher concentrations of sST2 were predictive for higher mortality [10]. As nonsurvivors in our series had slightly lower IL-33 levels simultaneously with significantly higher sST2 levels, our findings are consistent with the idea of higher “binding rates” of IL-33.

The incidence of infections is the greatest within the first 3 months after LVAD implantation, affecting about 25% of the patients [21]. Accordingly, we found a high incidence of predominantly Gram-positive bloodstream infections, presumptively related to central venous line or lower respiratory tract infections. Surprisingly, we observed a significantly higher frequency of pulmonary *Candida* colonisation in nonsurvivors. Although the sST2 levels in patients with pulmonary *Candida* colonisation were almost identical to those of non-Candida isolated patients during the first 3 days, they were persistently higher after day 4. This time point also coincided with the first positive cultures of *Candida* species isolated from the BAL of patients. Therefore, one might speculate that the presence of *Candida* species in the lungs of nonsurvivors is a result of reduced fungicidal activity. On the other hand, inflammatory cytokines stimulate lung alveolar epithelial cells to produce sST2 via an NF-*κ*B-dependent mechanism [11]. Therefore, it seems also plausible that *Candida* species stimulate lung alveolar epithelial cells to release sST2. The fungicidal activity of neutrophils and macrophages depends on Th-1 cell responses and IFN*γ* production [22]. ST2 deficiency has recently been shown to shift the immune balance towards a Th-1 type, thereby improving the outcome of patients with *Staphylococcus aureus*-induced septic arthritis [23]. Conversely, enhanced sST2 levels facilitate infections by promoting a systemic anti-inflammatory state that results in “endogenous immunosuppression” [10]. Even though the mechanism of sST2-induced suppression of proinflammatory cytokines is not well understood, ST2 has been shown to prevent the binding of the NF*κ*B complex to the IL-6 promoter as well as I*κ*B degradation in THP-1 cells. As the nuclear factor- (NF)-*κ*B is important for the expression of inflammatory cytokines, sST2 negatively affects IL-6 production [24].

Undeniably, the concept of pulmonary candida infection/colonisation continues to be very controversial and our data cannot discriminate an invasive fungal infection or a colonisation of the respiratory tract. Nevertheless, there are some indices from other studies that LVAD implantation results in an aberrant state of T cell activation, heightened susceptibility of CD4 T cells to activation-induced cell death, progressive defects in cellular immunity, and higher risk of Candida infections 3 months after LVAD implantation compared to heart failure patients under standard medical care [4, 25]. However, the role of sST2 and *Candida* infections in the pathophysiological mechanisms leading to more severe outcomes after LVAD implantation requires further investigation.

One major question that emerges from our study is what the primary source of sST2 is. sST2 is elevated in mechanically overloaded cardiomyocytes [26], suggesting that sST2 is induced under conditions of myocardial volume overload [27]. Besides cardiac myocytes, alveolar pneumoepithelial cells are also an important source of sST2 [11]. These cells can secrete sST2 spontaneously. The lungs, with their large surface area, are a rather plausible source of the high concentrations of sST2 seen on day 1 [11]. Our hemodynamic data demonstrate that LVAD is an effective therapy, at least in the short term, in end-stage heart failure. Therefore, it is unlikely that the massive sST2 release on day 1 is a result of exacerbating mechanical stress on cardiomyocytes or backward failure on pulmonary alveolar cells. It seems to be more likely that the sST2/IL-33 axis in the early phase after LVAD implantation is dominated by an “immunological signal” of sST2-enhancing cytokines caused by the surgical trauma and contact of patient's blood to compounds of heart-lung-machine (HLM) or extracorporeal membrane oxygenator (ECMO).

In contrast, the persistently elevated sST2 levels in 1-year nonsurvivors over the entire observation period may be interpreted as “background signal noise,” signaling the activation of profibrotic remodeling and a higher degree of disease progression [13, 15]. Caselli and coworkers found that myocardial expression of the ST2 gene is significantly enhanced one month after LVAD implantation, whereas levels of sST2 remained unaffected and IL-33 levels dropped significantly [28]. In contrast, other authors describe a significant decline of sST2 compared to baseline more than 60 days after LVAD implantation [13, 29]. Our data showed that sST2 levels return to baseline after day 2, remaining higher in 1-year nonsurvivors than in survivors. Interestingly, we did not see a recovery of sST2 to reference values (<35 ng/ml) until the end of the third postoperative week. This suggests that the sST2 axis is persistently “upregulated” in heart failure patients and even more so in 1-year nonsurvivors.

The persistently enhanced sST2 levels observed in nonsurvivors may express a higher degree of disease progression by antagonizing IL-33-mediated beneficial effects [15].

According to the literature, the most common causes of death after LVAD implantation are neurologic dysfunction (19% of the patients)—mainly from hemorrhagic and ischemic stroke—and multiorgan failure (15% of the patients). Major infection accounts for 8% of all deaths [2]. The incidence of sepsis causing death in our series may be underestimated, owing to more predominant causes of death that coincide with infection, such as bleeding and stroke. Our observations are in line with previous data, highlighting the still unsolved problems in the long-term support of heart failure patients implanted with LVAD.

### 4.1. Limitations

Our study has some limitations. Given the small sample size, the results may not be generalizable to other LVAD cohorts. Moreover, since the local ethics committee required an informed consent to be signed before LVAD implantation, we may have inadvertently biased our results by excluding >50% of all patients, most of them with worse clinical status (i.e., patients supported by ECMO and/or intubated before LVAD implantation who could not sign the informed consent). Finally, a microbiological analysis was not performed systematically for all patients, possibly introducing a bias in the results.

A further point of criticism is the heterogeneity concerning the implanted LVAD system. The distribution of 50% HVAD and 50% HM II implants in this series was not planned and is a result of the surgeons' discretion in the choice of the different types of LVAD manufacturer and designs. However, reflecting the actual available data of the INTERMACS registry of 2019, our data confirm the actual trend of almost equally represented axial flow (49%) and centrifugal flow (51%) LVAD implants by the year 2017 [2]. Furthermore, the type of device in our series interacted neither with outcome nor significantly with the time course of sST2.

## 5. Conclusion

Our findings revealed that patients with high sST2 levels have higher odds of worse outcomes, irrespective of therapeutic intent or device type. The findings of this study may have important implications for patients considering LVAD placement. As to what extent the circulating levels of the biomarker sST2 may help to identify patients at risk for worse outcomes has to be evaluated in larger trials. This early immune response should be taken into consideration when assessing the risks and benefits of LVAD implantation.

## Figures and Tables

**Table 1:** Baseline characteristics of the study population (*n* = 20).

Variables			
	1-year survivors (*n* = 13)	1-year nonsurvivors (*n* = 7)	*p* value
Age (y)	59 (56-69)	64 (60-69)	0.21^a^
BMI (kg/m^2^)	24.7 (22.7-29)	24.9 (22.8-34.4)	0.55^a^
Female	4 (30.8)	2 (28.6)	0.92^b^
Ntpro-BNP (pg/ml)	6094 (3856-6818)	5492 (1918-15000)	0.84^a^
INTERMACS level			0.83^b^
I	1 (7.6)	1 (14.3)	
II	3 (23)	1 (14.3)	
III	7 (53.8)	3 (42.9)	
IV	2 (15.3)	2 (28.6)	
Therapeutic intent			
Bridge to decision	0 (0)	1 (14.3)	
Bridge to candidacy	8 (61.5)	4 (57.1)	
Bridge to transplant	1 (7.7)	0 (0)	
Destination therapy	4 (30.8)	2 (28.6)	
Diabetes			0.37^b^
NIDDM	0 (0)	1 (14.3)	
IDDM	4 (30.8)	2 (28.6)	
History of arterial hypertension	9 (69.2)	4 (57.1)	0.58^b^
CHA2DS2-VASc score	3 (3-4)	3 (2-5)	1^a^
Malignant arrhythmia	2 (15.3)	1 (14.3)	0.94^b^
COLD	6 (46.2)	1 (14.3)	0.32^b^
Renal insufficiency (including patients on HF)	9 (69.2)	3 (42.9)	0.35^b^
Creatinine (mg/dl)	1.25 (0.91-1.4)	1.04 (1.01-1.8)	0.6^a^
Left ventricular ejection fraction (%)	20 (15-25)	20 (15-25)	0.93^a^
Systolic PAP (mmHg)	59 (45-67)	47 (38-57)	0.21^a^
Types of cardiomyopathy (CMP)			0.58^b^
Ischemic CMP	9 (69.2)	4 (57.1)	
Dilated CMP	4 (30.8)	3 (42.9)	

Values are medians with interquartile ranges (IQRs) or absolute numbers (percentages); ^a^Mann–Whitney *U* test, ^b^Fisher exact or chi-square test. BMI = body mass index; BNP = brain natriuretic peptide; INTERMACS = Interagency Registry for Mechanically Assisted Circulatory Support; NIDDM = non-insulin-dependent diabetes mellitus; CHA2DS2-VASc score = congestive heart failure, hypertension, age > 75, diabetes, prior, stroke/transient ischemic attack; IDDM = insulin-dependent diabetes mellitus; COLD = chronic obstructive lung disease; HF = hemofiltration; PAP = pulmonary artery pressure.

**Table 2:** Procedural data (*n* = 20).

Variables			
	1-year survivors (*n* = 13)	1-year nonsurvivors (*n* = 7)	*p* value
Duration of LVAD implantation (min)	263 (250-342)	327 (276-370)	0.25^a^
Duration of anesthesia (min)	365 (340-420)	457 (369-478)	0.21^a^
Surgical access			1^b^
Sternotomy	5 (38.5)		
Minimal invasive	8 (61.5)		
Circulatory support			0.44^b^
ECMO	2 (15.4)	0 (0)	
HLM	8 (61.5)	6 (85.7)	
Off-pump	3 (23.1)	1 (14.3)	
Type of device			0.35^b^
HM II	5 (61.5)	5 (71.4)	
HVAD	8 (38.5)	2 (28.6)	

Medians with interquartile ranges (IQRs) or absolute numbers (percentages); ^a^Mann–Whitney *U* test, ^b^Fisher exact or chi-square test. LVAD = left ventricular assist device; ECMO = extracorporeal membrane oxygenation; HLM = heart-lung machine; HM II = Thoratec® HeartMate II®; HVAD = HeartWare®.

**Table 3:** Pathogens isolated from patients during hospital stay.

Patient ID	Bacteria isolated from blood	Fungal pathogens	
		Species	Site(s) of detection
2	*Corynebacterium amycolatum*	*Candida albicans*	Urine culture
5	No pathogen isolated	*Candida albicans*, *Candida glabrata*	Urine culture, wound, BAL
6	*Staphylococcus epidermidis*	*Candida glabrata*	Urine culture
7	No pathogen isolated	*Candida lusitaniae*	BAL
8	No pathogen isolated	*Candida albicans*	Urine culture, BAL
9	No pathogen isolated	*Candida parapsilosis*	BAL, central venous line
12	No pathogen isolated	*Candida albicans*	BAL
16	*Corynebacterium*, *Staphylococcus capitis*	*Candida albicans*	Urine culture, BAL
17	*Staphylococcus haemolyticus*	*Candida albicans*, *Candida glabrata*	BAL
19	*Staphylococcus epidermidis*	*Candida glabrata*	Feces
20	No pathogen isolated	*Candida albicans*	Wound

The table shows all patients with positive bacteria or fungal pathogens in blood culture or at other sites; BAL = bronchoalveolar lavage; ID = identification number; VRE = vancomycin-resistant enterococcus.

**Table 4:** Causes of death (*n* = 7) and time to death.

Stroke (after 86 days)
Intracranial bleeding (after 236 days)
Liver failure (after 39 days)
MOF (after 175 days)
Sepsis (after 229 days)
Intestinal ischemia after pump thrombosis (after 31 days)
Hypoxic encephalopathy (therapy terminated) (after 31 days)

## Data Availability

Underlying data supporting the results of our study can be found in the Patient Data Management System of the Vienna General Hospital.
